# Ocean acidification and responses to predators: can sensory redundancy reduce the apparent impacts of elevated CO_2_ on fish?

**DOI:** 10.1002/ece3.684

**Published:** 2013-09-02

**Authors:** Oona M Lönnstedt, Philip L Munday, Mark I McCormick, Maud C O Ferrari, Douglas P Chivers

**Affiliations:** 1 ARC Centre of Excellence for Coral Reef Studies and School of Marine and Tropical Biology, James Cook UniversityTownsville, QLD, 4811, Australia; 2 Department of Biomedical Sciences, WCVM, University of SaskatchewanSK, S7N 5B4, Canada; 3 Department of Biology, University of SaskatchewanSK, S7N 5E2, Canada

**Keywords:** Chemical alarm cues, ocean acidification, predator, prey, sensory redundancy, visual cues

## Abstract

Carbon dioxide (CO_2_) levels in the atmosphere and surface ocean are rising at an unprecedented rate due to sustained and accelerating anthropogenic CO_2_ emissions. Previous studies have documented that exposure to elevated CO_2_ causes impaired antipredator behavior by coral reef fish in response to chemical cues associated with predation. However, whether ocean acidification will impair visual recognition of common predators is currently unknown. This study examined whether sensory compensation in the presence of multiple sensory cues could reduce the impacts of ocean acidification on antipredator responses. When exposed to seawater enriched with levels of CO_2_ predicted for the end of this century (880 μatm CO_2_), prey fish completely lost their response to conspecific alarm cues. While the visual response to a predator was also affected by high CO_2,_ it was not entirely lost. Fish exposed to elevated CO_2,_ spent less time in shelter than current-day controls and did not exhibit antipredator signaling behavior (bobbing) when multiple predator cues were present. They did, however, reduce feeding rate and activity levels to the same level as controls. The results suggest that the response of fish to visual cues may partially compensate for the lack of response to chemical cues. Fish subjected to elevated CO_2_ levels_,_ and exposed to chemical and visual predation cues simultaneously, responded with the same intensity as controls exposed to visual cues alone. However, these responses were still less than control fish simultaneously exposed to chemical and visual predation cues. Consequently, visual cues improve antipredator behavior of CO_2_ exposed fish, but do not fully compensate for the loss of response to chemical cues. The reduced ability to correctly respond to a predator will have ramifications for survival in encounters with predators in the field, which could have repercussions for population replenishment in acidified oceans.

## Introduction

The concentration of carbon dioxide (CO_2_) in the atmosphere is rising at a rate unprecedented for millions of years, due to the release of CO_2_ from fossil fuel burning, cement production, and land use changes by humans (Doney et al. 2009). Atmospheric CO_2_ is now 400 ppm (Dlugokencky and Tans 2013), higher than any time in the past 800,000 years (Luthi et al. 2008), and could exceed 900 ppm by the end of the century if the current emissions trajectory is maintained (Meinshausen et al. 2011; Peters et al. 2012). One of the consequences of rising atmospheric CO_2_ concentrations is the increased absorption of CO_2_ in the ocean. Here, it reacts with seawater, causing a reduction in the concentration of carbonate ions and lowering seawater pH, a process known as ocean acidification (Raven et al. 2005). The partial pressure of CO_2_ (Pco_2_) in the ocean also increases with increasing atmospheric CO_2_ because the ocean surface is at approximate gas equilibrium with the atmosphere (Doney 2010). These changes to ocean chemistry have been shown to affect fundamental biological processes, such as metabolism, growth, calcification, reproduction, and behavior, in a wide range of marine organisms (Fabry et al. 2008; Widdicombe and Spicer 2008; Doney et al. 2009; Kroeker et al. 2010; Briffa et al. 2012). However, the potentially interacting effects of ocean acidification on multiple biological traits, and the effects on ecological interactions among organisms, remain poorly understood (Fabry et al. 2008; Hendriks et al. 2010; Kroeker et al. 2013). Anticipating the responses of marine organisms to rising CO_2_ levels and ocean acidification is a crucial test case for evolutionary ecologists.

Recent studies show that exposure to elevated CO_2_ causes fish to fail to respond to ecologically important chemical cues including homing odors (Munday et al. 2009) and predation cues (Dixson et al. 2010; Ferrari et al. 2011a). Munday et al. (2010) and Ferrari et al. (2011b) both found that larval fish (*Pomacentris wardi* and *Pomacentris chrysurus*) raised in seawater enriched with levels of CO_2_ predicted for the end of this century dramatically altered their behavior and displayed higher mortality compared to fish raised in current-day seawater. Fish exposed to elevated CO_2_ levels have also been found to have impaired auditory abilities (Simpson et al. 2011) and reduced behavioral lateralization (Domenici et al. 2012) providing evidence that high CO_2_ directly affects brain function in juvenile fish (Nilsson et al. 2012). One study has demonstrated that ocean acidification will also affect recognition or cognitive processing of visual information. Ferrari et al. (2012) found that larval damselfish raised in high-CO_2_ seawater responded differently to the sight of a large nonpredatory fish (a spiny Chromis, *Acanthochromis polyacanthus*) to which the prey was unfamiliar. Fish exposed to current-day levels of CO_2_ reacted to *A. polyacanthus* with antipredator behaviors indicating that the prey may show neophobic responses to any large fish, regardless of whether they pose a threat (Brown et al. 2013). Whether ocean acidification will impair visual recognition of prey to common predators is currently unknown.

A recent study by Devine et al. (2012) found that there were differential effects of CO_2_ on different senses and that one sense might overcome the impairment of another sense. Such sensory redundancy could reduce the effects of high CO_2_. In Devine et al.'s (2012) study, three species of damselfish that depend on both olfactory and visual cues to find appropriate settlement habitats retained the ability to select their preferred habitat although their olfactory sense had been impaired. This suggests that at least some fish will rely on other senses to compensate for the loss of one sense. Here, we investigated the effects of elevated CO_2_ on responses of fishes to visual, chemical, and visual cues paired with chemical cues of predators. Specifically, we were interested in experimentally testing whether appropriate responses to visual information could compensate for impaired olfactory antipredator senses that are commonly reported in damselfish.

The early life stages of many animals, including fishes, are extremely vulnerable to predation (Almany and Webster 2006). Due to their development in the plankton, settlement stage reef fish arrive to habitat patches that contain predators never before encountered. At this time, individuals would benefit from possessing a preprogrammed (i.e., innate) sensory response to a predation threat, be it visual or olfactory. There are many cases of fishes displaying innate responses to skin extracts of injured conspecifics (Chivers and Smith 1998; Lönnstedt and McCormick 2011) and odor cues emitted by their natural predators (Hawkins et al. 2004; Dixson et al. 2010). However, limited research has investigated if naïve prey fish display an innate response to the visual cues of predators (for an exception see Coss 1979). Highly diverse environments, such as coral reefs, contain vast numbers of fish of different colors, sizes, and body shapes (Marshall et al. 2003). Here, the ability to visually discriminate between predators and nonpredators should be of crucial importance for naïve prey. While it is known that ocean acidification impairs the ability of prey fish to detect olfactory signposts of risk, it is unknown whether visual information may partially compensate for the lack of olfactory abilities, and thus help maintain population replenishment in acidified oceans.

The current study aimed to determine how prey fish (ambon damselfish, *Pomacentrus amboinensis)* respond to the separate and combined effects of olfactory and visual cues of predators when exposed to a CO_2_ level (880 μatm CO_2_) projected for the surface ocean by the end of this century (Doney 2010; Meinshausen et al. 2011). We specifically chose to study ambon damselfish because they appear to be the most sensitive of the four species of damselfishes to CO_2_-induced impairment of their olfactory sense (Ferrari et al. 2011b). In order to test whether appropriate responses to visual cues could compensate for the loss of response to chemical cues, we conducted three experiments. The first experiment was designed to test how naïve (with no prior experience of predators) fish exposed to elevated Pco_2_ (880 μatm) respond to damage-released skin extracts of conspecifics. Secondly, we tested whether responses to visual cues of a common predator were impaired in response to elevated Pco_2_. The final experiment examined if fish retained an antipredator response when exposed to visual and chemical indicators of risk simultaneously, testing whether the visual sense could overcome impairment of the olfactory sense.

## Methods

### Study species and sampling

All experiments were carried out at Lizard Island Research Station (14°40'S, 145°28'E), northern Great Barrier Reef, Australia in October–November 2010. Ambon damselfish, *P. amboinensis*, are a common component of reef fish communities around the Indo-Pacific. The predatory dottyback, *Pseudochromis fuscus*, is known to specialize and feed on newly settled fish during the recruitment season (Feeney et al. 2012) and was therefore used as the model predator for all experiments. *P. fuscus* is common cryptic predator on coral reefs and is found in habitats occupied by *P. amboinensis*. All fish were collected at the end of their larval phase (while naïve to reef-based, bottom-dwelling predators) using light traps that had been moored overnight off the reefs at Lizard Island. Fish were brought back to the research station and transferred into 35-L flow-through seawater aquaria (in groups of 20–30 fish) maintained at one of two CO_2_ concentrations for four consecutive days (12L:12D photoperiod). Previous studies have shown that exposing fish to elevated CO_2_ for 4 days leads to identical behavioral impairment as fish that have been exposed to high CO_2_ since hatching (Munday et al. 2010). Furthermore, longer term exposure does not produce any acclimation of behavioral responses, with the behavior of juveniles similarly impaired after 4 days and 4 weeks (Munday et al. 2013). Fish were fed ad libitum three times daily with newly hatched brine shrimp, *Artemia sp*. *P. fuscus* were collected on the fringing reefs around Lizard Island on SCUBA using a dilute clove oil anesthetic and hand nets. Captured fish were placed in 10-L plastic bags and transported to the research station where they were held in 30-L flow-through seawater tanks. Each plastic holding tank had a 2 cm layer of sand at the bottom and contained several plastic tubes that served as shelters.

### Ocean acidification system

*Pomacentrus amboinensis* were held a minimum of 96 h in replicate aquariums supplied with a constant flow of either control seawater (440 μatm CO_2_) or water enriched with carbon dioxide (880 μatm CO_2_) (Table 1). *P. amboinensis* were kept in treatment for a minimum of 96 h as previous studies have demonstrated that this is sufficient time for juvenile fish to be behaviorally affected by elevated CO_2_ and longer exposure does not further alter the behavioral changes associated with CO_2_ treatment (Munday et al. 2010). Effectively, fishes treated for 96 h behave the same as fishes reared from hatching in seawater with elevated CO2 (Munday et al. 2010), as described above. CO_2_ treatments were maintained by CO_2_ dosing to a set pH_NBS_. Seawater was pumped from the ocean into 2 × 60 L sumps where it was diffused with ambient air or CO_2_ to achieve a pH of ∼8.15 (current day), or ∼7.89 (a value which is expected to be reached by the end of this century and CO_2_ emissions continue along the current trajectory). To maintain pH at the desired level, a pH controller (Tunze Aquarientechnik, Germany) was attached to the CO_2_-treated sump. A solenoid injected a slow stream of CO_2_ into a powerhead at the bottom of the sump whenever the pH of the seawater rose above the set point. The powerhead dissolved CO_2_ into the seawater while simultaneously serving as a vigorous stirrer. Equilibrated seawater from each sump was supplied at a rate of ∼500 mL/min to four replicate 35-L aquariums, each housing a group of larval fishes. To maintain oxygen levels and the required Pco_2_ levels, aquaria were individually aerated with unmanipulated air or CO_2_-enriched air (∼880 ppm). The concentration of CO_2_-enriched air was controlled by a scientific-grade pressure regulator and precision needle valve and measured continuously with an infrared CO_2_ probe (Vaisala GM70). Temperature and pH_NBS_ of each aquarium was measured each morning and afternoon using a HQ40d pH meter (Hach, Loveland, CO) calibrated with fresh buffers. Total alkalinity of seawater was estimated by Gran titration from water samples taken twice weekly from each CO_2_ treatment. Alkalinity standardizations performed before processing each batch achieved accuracy within 1% of certified reference material from Prof. A. Dickson (Scripps Institution of Oceanography). Average seawater Pco_2_ was calculated using these parameters in the program CO2SYS and using the constants of Mehrbach et al. (1973) refit by Dickson and Millero (1987). Estimated seawater parameters are shown in Table 1.

**Table 1:** Mean (±SD) seawater parameters in the experimental system

pH_NBS_	Temp°C	Salinity ppt	TA μmol/kg SW	Pco_2_ μatm
8.15 (0.04)	27.66 (0.98)	35	2269.66 (15.01)	440.53 (44.46)
7.89 (0.06)	27.74 (0.99)	35	2261.23 (14.92)	879.95 (140.64)

Temperature, pH salinity, and total alkalinity (TA) were measured directly. Pco_2_ was estimated from these parameters using CO2SYS.

### Experimental protocol

The experimental protocol is described as three separate experiments because a number of different controls were required to experimentally examine the response of fish from the elevated CO_2_ treatment to chemical or visual stimuli. All experiments were conducted on random subsets of *P. amboinensis* which had been collected at a similar time and location, making comparisons across experiments valid. The same number of fish from both the control and CO_2_ treatments was tested on a given day, and the order of testing was randomized.

Following the CO_2_ conditioning, individual *P. amboinensis* were transferred into transparent 15-L aquaria (38 × 24 × 27 cm) with a constant flow of fresh seawater and allowed to acclimate overnight. Juvenile damselfish are known to retain their impaired behavioral response for a period of 48 h after being returned to ambient seawater, and this response is no different to fish tested in elevated CO_2_ water within the 48-h window (Munday et al. 2010; Nilsson et al. 2012). Each aquarium was covered on three sides by black plastic with one long side having a 3 × 3 cm grid drawn on it. A single airstone was placed at the back corner of each tank with two 1.5-meter-long plastic tubes fixed to the airline (one for the injection of food, and one for the injection of the experimental stimuli) allowing for rapid dispersal of extracts in the aquaria. Each tank contained a 2 cm layer of coral sand on the bottom and a live coral habitat (bushy hard coral, *Pocillopora damicornis)* along the short side of the aquaria creating a vertical shelter.

Prior to the start of the trial, the water flow was stopped and 5 ml of *Artemia sp* (∼550 *Artemia*) was added to the aquaria to stimulate feeding. The behavior of a single *P. amboinensis* was recorded for a 4-min prestimulus period. Immediately following the prestimulus period, food was injected again followed by one of the seven different stimuli (depending on the experiment as described below), and the fish's behavior was recorded for a further 4 min. Three behaviors were categorized and recorded: foraging, activity, and shelter use. Foraging was recorded as the total number of feeding strikes, activity level was quantified as the number of times a fish crossed a line on the grid, and shelter use was recorded as the total amount of time a fish spent in shelter.

### Experiment 1: Does elevated CO_2_ impair responses to chemical alarm cues?

Control and elevated-CO_2_-treated *P. amboinensis* were tested to determine whether they respond to chemical cues released from damaged conspecifics. We also tested for a behavioral response to extracts from damaged heterospecifics (*Apogon doederlini*) and a saltwater control. The heterospecific skin extract allowed us to establish whether juvenile *P. amboinensis* have a generalized behavioral response to any injured fish, while the saltwater stimulus served as an additional disturbance control. To prepare the alarm cues, we sacrificed the donor fish using cold shock. The flank of each recruit was then superficially cut six times. The total cue area was rinsed with 10 mL of saltwater and filtered through filter paper (47 mm Ø) prior to being used in the experiment.

### Experiment 2: Does elevated CO_2_ impair responses to the sight of a predator?

To test whether naïve *P. amboinensis* respond to the visual stimuli of a predator, we assessed the change in behavior of control and elevated-CO_2_-treated fish upon presentation of a predator (*P. fuscus*). During each trial we introduced 5 mL of *Artemia sp*. and then quantified the behavior of the fish for 4 min as in experiment 1. Following the prestimulus period, a watertight plastic bag (15 × 23 cm) containing the predator was carefully lowered into the aquaria on the opposite side of the coral shelter. After a 30-sec stimulus introduction period, a further 5 mL of *Artemia sp*. was added to the aquarium and the behavior of the focal fish was quantified for a further 4 min. Fish were also exposed to bags containing (1) a nonpredatory fish (*Amblygobius phalanea*) and (2) an empty bag controlling for changes resulting from the experimental procedure. To control for a response to the visual stimulus of any fish, we used the herbivorous goby, as it is a similar size and shape to *P. fuscus* but nonpredatory.

### Experiment 3: Does sensory redundancy reduce the apparent impact of elevated CO_2_?

In this experiment, control and elevated-CO_2_-treated *P. amboinensis* were exposed to (1) chemical alarm cues alone, (2) visual predator cues alone, or (3) a combination of visual and chemical cues. The magnitude of the response of *P. amboinensis* to the paired chemical and visual stimuli was then compared to the magnitude of response to chemical and visual cues in isolation. In this last experiment, we added a fifth behavioral measure: bobbing. Bobbing is a common antipredator behavior in juvenile damselfish and consists of raising the anterior portion of the body, followed by a rapid descent, which is repeated several times (Smith and Smith 1989; Ferrari et al. 2012).

### Statistical analysis

For all of the experiments, the difference in behavior between the prestimulus and poststimulus periods was calculated and used for analysis. Differences between the control and elevated CO_2_ treatment were examined using a one-way multivariate analysis of variance (MANOVA). A two-factor MANOVA was then employed to examine whether the behavior differed between fish from the two CO_2_ treatments in response to the cue stimulus type (olfactory, visual or a combination of the two). The behavioral variables included in the analysis were feeding strikes, activity level, and time spent in shelter (s). To further explore the nature of significant differences found by the MANOVA, univariate ANOVAs were used and significant differences were further examined using Tukey's Honestly Significant Difference (HSD) means comparison tests. The assumptions were examined and time spent in shelter was Log10(*x* + 1) transformed.

Log-linear models were used to examine how threat sources (conspecific skin extract, visual predator, or both) and CO_2_ treatment (elevated and current day) affected the occurrence of bobbing behavior in the third experiment. Models were constructed to test five specific hypotheses concerning bobbing frequency: (1) independent of treatment or cue; (2) dependent on cue; (3) dependent on treatment; (4) dependent on both treatment and cue; and (5) dependent on an interaction between treatment and cue. The models were fitted to the observed data in increasing order of complexity until there was no significant improvement in the goodness-of-fit statistic (likelihood ratio chi-square) from one model to the next. All statistics were undertaken using Statistica (v 10).

## Results

### Experiment 1: Does elevated CO_2_ impair responses to chemical alarm cues?

**Figure 1:**
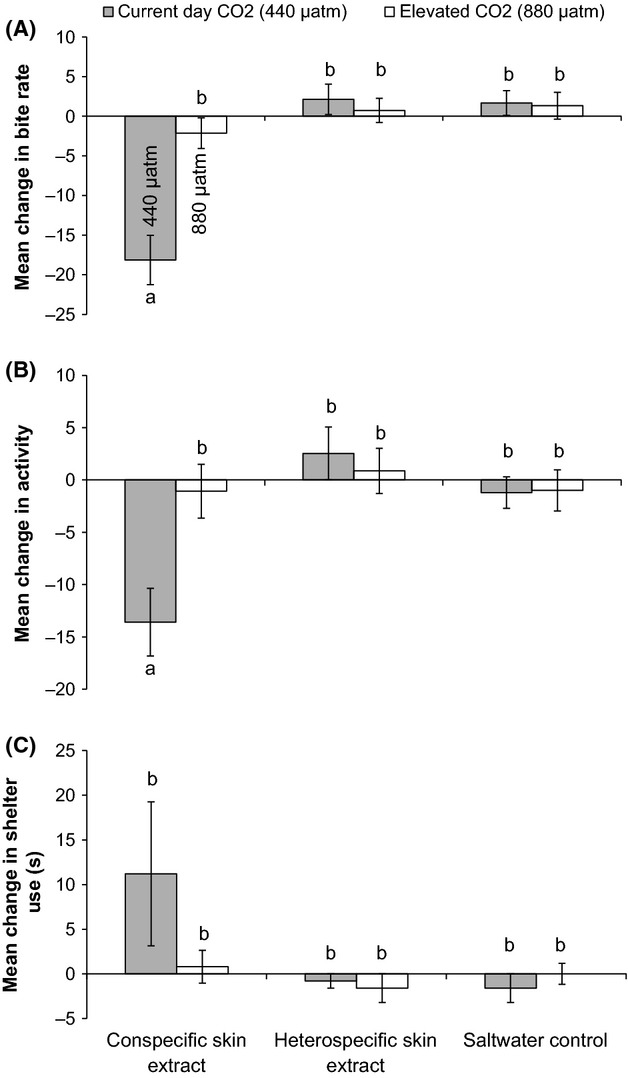
Mean change (±SE) of behavior in (A) feeding strikes, (B) activity level, and (C) time spent in shelter (s) by naïve *Pomacentrus amboinensis* when exposed to conspecific skin extracts, heterospecific skin extracts, or a saltwater control between the pre- and poststimulus period for fish exposed to two different CO_2_ concentrations. Letters above or below bars represent Tukey's HSD groupings of means.

There was no effect of elevated CO_2_ levels on the behavior of fish during the prestimulus period (Pillai's Trace: *F*_6,166_ = 0.08, *P* > 0.5). However, there was a strong influence of elevated CO_2_ on the response of fish to conspecific skin extracts (Pillai's Trace: *F*_6,166_ = 0.23, *P* = 0.002; Fig. 1). *P. amboinensis* from the elevated CO_2_ treatment did not elicit an antipredator response when exposed to the conspecific skin extracts (Fig. 1A and B). In contrast, prey exposed to current-day CO_2_ treatment displayed a typical threat response to conspecific chemical alarm cues, greatly reducing both foraging (*F*_2,84_ = 11.5, *P* < 0.001; Fig. 1A) and activity rates (*F*_2,84_ = 5.2, *P* = 0.007; Fig. 1B) compared to those exposed to heterospecific skin extracts or to saltwater control. Fish exposed to either elevated or current-day CO_2_ treatments did not increase time spent in shelter upon the injection of conspecific skin extracts (*F*_2,84_ = 1.6, *P* = 0.2; Fig. 1C). Although there was a tendency for fish from the current-day CO_2_ treatment to spend more time in shelter following injection of conspecific skin extracts, this was not statistically significant due to the large variation in responses among individuals (Fig. 1C).

### Experiment 2: Does elevated CO_2_ impair responses to the sight of a predator?

**Figure 2:**
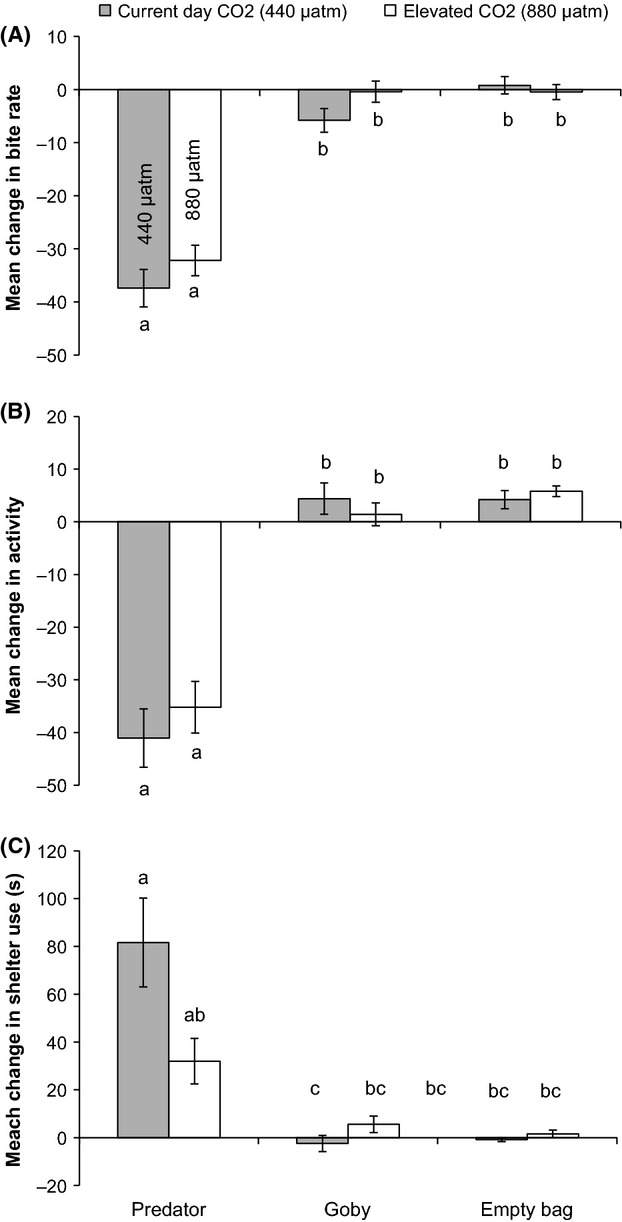
Mean change (±SE) in behavior in (A) feeding strikes, (B) activity level, and (C) time spent in shelter (s) by naïve *Pomacentrus amboinensis* when exposed to the sight of a common predator, *Pseudochromis fuscus*, a herbivorous goby (*Amblygobius phalanea*) or an empty bag control between the pre- and poststimulus period for fish exposed to two different CO_2_ concentrations. Letters above or below bars represent Tukey's HSD groupings of means.

There was no effect of elevated CO_2_ levels on the behavior of fish during the prestimulus period (Pillai's Trace: *F*_6,166_ = 0.43, *P* > 0.5). Overall there was no effect of elevated CO_2_ on the visual response of prey to the predator compared to the two controls (Pillai's Trace: *F*_3,164_ = 0.14, *P* = 0.4; Fig. 2). Univariate ANOVAs revealed that there was a significant reduction in feeding rate (*F*_2,84_ = 1.3, *P* = 0.3; Fig. 2A) and activity (*F*_2,84_ = 0.9, *P* = 0.4; Fig. 2B) when *P. amboinensis* juveniles were exposed to the sight of a predator irrespective of the CO_2_ treatment. Time spent in shelter increased in fish exposed to the visual sight of a predator compared with the two experimental controls (*F*_2,84_ = 4.6, *P* = 0.01; Fig. 2C); however, the response was not identical between CO_2_ treatments. Fish from the current-day treatment significantly increased shelter use upon being presented with the visual cue of a predator control, whereas there was no significant difference in shelter use in the fish exposed to elevated CO_2_ levels and the two controls (heterospecific skin extract and saltwater). The mean change in shelter time of fish from the elevated CO_2_ treatment was intermediate to the fish from the current-day treatments and the two controls, suggesting that there were some effect of the high CO_2_ on visual response to the predator (Fig. 2C).

### Experiment 3: Does sensory redundancy reduce the apparent impact of elevated CO_2_?

**Figure 3:**
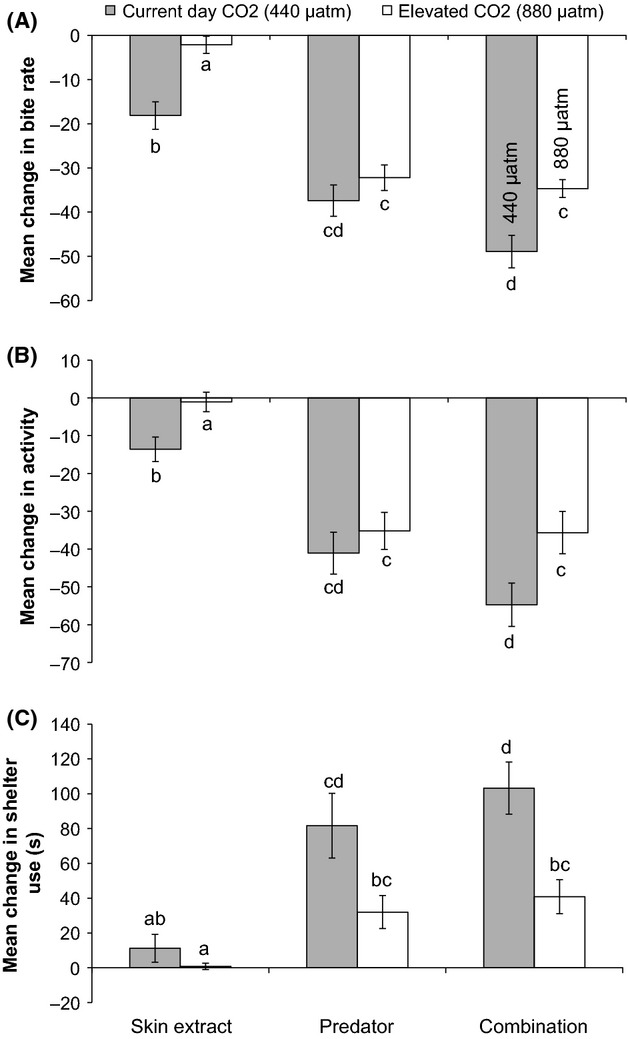
Mean change (±SE) in behavior in (a) feeding strikes, (b) activity level, and (c) time spent in shelter (s) by naïve *Pomacentrus amboinensis* when exposed to conspecific skin extracts, the sight of a common predator, *Pseudochromis fuscus*, or a combination of the two between the pre- and poststimulus period for fish exposed to two different CO_2_ concentrations. Letters above or below bars represent Tukey's HSD groupings of means.

As observed in the previous two experiments, there was no effect of elevated CO_2_ levels on the behavior of fish during the prestimulus period (Pillai's Trace: *F*_6,166_ = 0.48, *P* > 0.5). The MANOVA revealed that there was an interaction between CO_2_ treatment and cue source on the different antipredator responses of fish (Pillai's Trace: *F*_3,164_ = 0.69, *P* = 0.001; Fig. 3). In fish exposed to current-day conditions, the combined cue sources gave the strongest threat responses whereas olfactory cues alone gave the weakest reaction (Fig. 3). Fish exposed to elevated CO_2_ concentrations did not respond to skin extracts, and the magnitude of their response to combined cue sources did not differ from that elicited when exposed to visual cues alone. Post hoc tests revealed that prey from the current-day treatment exposed to both cue sources significantly reduced foraging and activity and increased time spent in shelter compared with the CO_2_-treated fish (Tukey's HSD: *P* < 0.05: Fig. 3).

**Figure 4:**
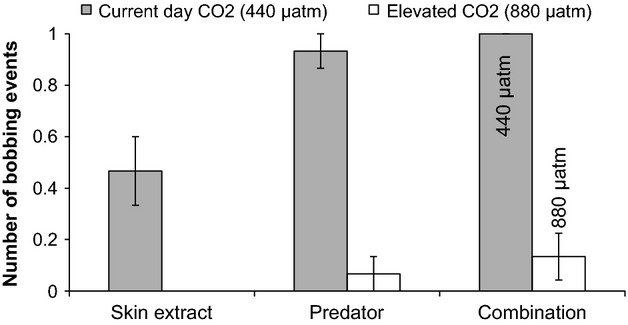
Mean number of times that *Pomacentrus amboinensis* displayed a bobbing event (±SE) when naïve fish from each of the two CO_2_ treatments were exposed to either conspecific skin extracts, the visual sight of a common predator, *Pseudochromis fuscus*, or a combination of the two. Fish exposed to the higher treatment (850 μatm) showed a significant decrease in the occurrence of this behavior as compared to fish exposed to current-day CO_2_ levels with all fish in this treatment responding to combined predator cues by bobbing (440 μatm).

CO_2_ treatment and cue exposure significantly affected the outcome of frequency of bobbing behavior by *P. amboinensis* (Table 2; model 4 was the best fit; Fig. 4). The inclusion of CO_2_ treatment in the model resulted in the greatest improvement in the fit of the log-linear model (Table 2; models 1 vs. 3) compared to the inclusion of cue in the model (Table 2; models 1 vs. 2). Therefore, although the outcomes of trials were dependent on both treatment and cue type, CO_2_ treatment had the greatest influence on frequency of bobs in *P. amboinensis*. Fish in the control treatment always responded with bobbing behavior to the simultaneous exposure of both cue sources, whereas fish in the elevated CO_2_ treatment bobbed significantly less than control fish, with only two of 15 fish displaying this type of antipredator behavior (Fig. 4).

**Table 2:** Determinant of bobbing frequency of *Pomacentrus amboinensis* when exposed to CO_2_ treatments (two levels) and predation risk cues (three levels)

Model	Likelihood ratio chi-square	df	Hypothesis: bobbing frequency is	df	Difference between models
(1) T × C + R	68.29***	6	Independent of treatment or cue		
(2) T × C + C × R	59.41***	3	Dependent on cue	3	1 and 2, 8.88*
(3) T × C + T × R	15.5**	4	Dependent on treatment	2	1 and 3, 52.79***
**(4)*****T ×*** ***C + T × R + C × R***	**0.63 NS**	**2**	**Dependence on both treatment and cue**	**2**	3 and 4, 14.87***
(5) T × C × R	0	0	Dependent on an interaction between treatment and cue	2	4 and 5, 0.63 NS

T, CO_2_ treatment (elevated, present day); C, cue (skin extract, visual, both); R, reaction (bobs, no bobs). *N* = 15.

Significance values comparing are indicated by: * = <0.05, ** = <0.01, *** = <0.005. Bold values indicate <0.0005.

## Discussion

This study suggests that both the visual and chemical antipredator systems of naïve prey are sensitive to changes in ocean acidification; however, the visually based behavioral responses are less affected than chemically based responses. Fish exposed to elevated CO_2_ completely failed to respond chemical alarm cues. While still responding to the sight of a common predator with reduced foraging and movement, *P. amboinensis* exposed to high CO_2_ displayed a delayed response to the piscivore spending less time in shelter. Furthermore, CO_2_-treated fish did not display the typical “bobbing” response common to damselfish when faced with a threat. It has been suggested that this “bobbing” behavior evolved as a means of pursuit deterrence; prey signal to the predator that they are aware of its presence, indicating to the predator that it is less likely to be successful in a strike (Smith and Smith 1989; Feeney et al. 2012). The lack of bobbing in fish exposed to elevated CO_2_ suggests that prey are capable of visually detecting a large shape, responding with increased vigilance, but may not label the shape as a predator. Alternatively, the fish could well recognize the predator, but choose not to initiate bobbing if this is an energetically costly behavior. Bobbing behavior is probably similar to that of stotting behavior in gazelles. Stotting is an honest signal of escape ability and only initiated by individuals that have the ability to escape from the predator (Caro 1986). The lack of a response to the herbivorous goby (visual control) in both the high- and low-CO_2_ treatments suggests that naïve fish are in fact able to visually discriminate between a threatening and nonthreatening fish. The response of the naïve prey exposed to acidified waters may simply represent a conservative neophobic response rather than an innate antipredator response. This apparent lack of ability to correctly categorize a predator, and thus assess risk, will have ramifications for survival in encounters with predators in the field (e.g., Munday et al. 2010).

In marine environments, visual and chemical cues are the key sources of information for assessing predation risk (Brown and Chivers 2006; Lönnstedt et al. 2012). Olfaction is often the first sense to alert a prey to the presence of a potential predator and once the prey is in the direct vicinity of the stimulus source vision takes over as the primary mode of predator detection (Brown and Chivers 2006).Visual cues are more reliable as they are fast, highly directional and provide accurate information on which informed behavioral decisions can be made, including predator size, speed of movement, and direction, as well as the likelihood of attack (Coss 1979; Helfman 1989). However, to accurately assess the level of threat that a predator poses, prey will often use multiple sensory cues as visual and chemical information provide complimentary information (Lima and Steury 2005; McCormick and Manassa 2008). Despite this, only few studies have compared the relative importance and balance of more than one stimulus. Given that predator avoidance behavior is modified based on the magnitude of threat, visual information may maintain antipredator behavior, even while olfaction is impaired. Although naïve prey exposed to current-day CO_2_ levels responded slightly more strongly to the simultaneous exposure to both sensory cues compared with the fish maintained in the elevated CO_2_ levels, there was no significant difference in the magnitude of responses in two common antipredator behaviors (activity and foraging) compared with the experimental controls when fish from both treatments were exposed to the sight of a predator. This suggests that the visual system of prey fish may be able to help mitigate some of the effects of the loss of the olfactory antipredator system, thus decreasing prey vulnerability to predators in acidified oceans.

Animals have been found to rely more strongly on one type of cue in environments where other cues necessary for predator detection are lacking. Fish have been found to rely more heavily on their chemical sense in situations where visual cues are limited. Stronger antipredator responses are found at night (Leduc et al. 2010) in turbid waters (Hartman and Abrahams 2000; Leahy et al. 2011) and in topographically complex habitats (M. I. McCormick, unpubl. data). Similarly, Chivers et al. (2001) found that in high visibility environments fish only respond to chemical cues if given in conjunction with the visual cue of a predator. In this study, we found that visual cues may be able to help compensate for the inability of fish to recognize threatening olfactory cues as fish still responded to the sight of *P. fuscus* with reduced foraging and movement. In the high clarity waters of coral reefs, it is crucial for prey fish to retain a visual response as they are surrounded by a multitude of different predators.

Our findings suggest that response of naïve prey to the predator *P. fuscus* is innate. Few studies have investigated the innate recognition of prey fish to the visual cue of piscivores. Coss (1979) found that naïve (reared in isolation; having received no previous predator cues) African jewel fish fry (*Hemichromis bimaculatus*) elicit evasive antipredator behaviors when exposed to the sight of a model with two front facing eyes (believed to be a widespread signal of danger) compared to a model with no eyespots. *P. fuscus* similarly has two frontally placed eyes, likely to label the fish as dangerous to new settlers. Coss's (1979) study taken together with the current study suggests that the ability of at least some fish to recognize predators appears to be predetermined. Katzenstein and Goren (2006) found that damselfish juveniles classify line drawings with “smiley faces” as nonthreatening and line drawings with “sad” faces as predators. It appears that fish are good at categorization of visual stimuli, thus it is possible that juveniles can generalize from their experiences with predators in the pelagic environment to predators on the reef. However, this visual recognition system appears to be impaired by ocean acidification as two of the crucial antipredator behaviors (bobbing and hiding) were absent in fish exposed to high-CO_2_ seawater. The lack of bobbing in naïve *P. amboinensis* exposed to acidified seawater suggests that the prey fail to recognize the predator as a threat, alternatively failing to initiate an appropriate antipredator response despite recognizing the piscivore as a threat, consequently spending less time in the security of shelter. This response could be attributed to CO_2_ interfering with the nervous system of fish. An elegant study carried out by Nilsson et al. (2012) found that similar CO_2_ levels (∼900 μatm) inhibit the sensory system of fish by interfering with GABA-A neurotransmitters, thereby affecting chemosensory, auditory, and visual abilities. If settlement stage fish are unable to determine the degree of possible threat due to reduced cognitive abilities, they may act in a cautious manner (as they would to any new stimulus) but not with the same intensity as to the presence of a known predator, as was found in this study.

Our results imply that some antipredator behaviors of fish to chemical and visual threats will be affected by ocean acidification. While the visual response is not entirely lost, the sense is affected by rising CO_2_ levels. Naïve prey exposed to higher CO_2_ concentrations did respond to the presence of a predator, but with a lower intensity than control fish, failing to retire to the safety of shelter. Their lack of appropriate behavioral responses to piscivores could pose a major problem when crepuscular and nocturnal predators are active, as vision is even more limited during these hours. This is the time of the day when the majority of mortality occurs, and a well-developed visual sense along with the olfactory sense plays a key role in the identification and avoidance of predators. The cost of missing a sign of a nearby predator can be fatal, as was seen in Munday et al. (2010) were larval fish (*P. wardi*) raised in seawater enriched with levels of CO_2_ predicted for the end of this century (∼850 μatm) displayed up to nine times higher mortality compared to fish raised in current-day seawater. These fish had access to all naturally available predation cues, indicating that a partially functioning visual system was insufficient to prevent dramatic increases in predation of high CO_2_ exposed fish. Additional field experiments are needed to determine whether the same would hold true for ambon damselfish.

Our study and previous studies have come to the same conclusion; the sensory systems and behavioral responses of fish will be severely affected in future acidified oceans. A key question is whether marine organisms will be able to adapt to the changing pH of the world's oceans (Kelly and Hofmann 2012; Munday et al. 2012a). We already know that some animals are no longer able to cope with environments they have spent thousands of generations specifically adapting to (Walther et al. 2002). And it is a cause for concern when the nervous system and instinctive behaviors of naïve prey are damaged or lost as a consequence of rising CO_2_ concentrations. Nevertheless, some studies have detected reduced impacts when several generations are exposed to the same high CO_2_ environment (Miller et al. 2012; Parker et al. 2012) and there could be the potential for selection of CO_2_ tolerant genotypes to occur over coming generations (Munday et al. 2012b). Whether differences in the severity of impacts to different sensory systems increases the potential for adaptation to a high CO_2_ environment remains to be seen, but should be a priority area for future research.
